# Cell Based Therapy for Type 1 Diabetes: Should We Take Hyperglycemia Into Account?

**DOI:** 10.3389/fimmu.2019.00079

**Published:** 2019-02-05

**Authors:** Anna Grohová, Klára Dáňová, Radek Špíšek, Lenka Palová-Jelínková

**Affiliations:** ^1^SOTIO a.s., Prague, Czechia; ^2^Department of Immunology, Second Faculty of Medicine, University Hospital Motol, Charles University in Prague, Prague, Czechia; ^3^Department of Pediatrics, Charles University in Prague, Second Faculty of Medicine, University Hospital Motol, Prague, Czechia

**Keywords:** dendritic cells, immune tolerance, cell-based therapy, diabetes mellitus, hyperglycemia

## Abstract

Diabetes mellitus is characterized by long standing hyperglycemia leading to numerous life-threatening complications. For type 1 diabetes mellitus, resulting from selective destruction of insulin producing cells by exaggerated immune reaction, the only effective therapy remains exogenous insulin administration. Despite accurate compliance to treatment of certain patients, transient episodes of hyperglycemia cannot be completely eliminated by this symptomatic treatment. Novel immunotherapeutic approaches based on tolerogenic dendritic cells, T regulatory cells and mesenchymal stem cells (MSCs) have been tested in clinical trials, endeavoring to directly modulate the autoimmune destruction process in pancreas. However, hyperglycemia itself affects the immune system and the final efficacy of cell-based immunotherapies could be affected by the different glycemic control of enrolled patients. The present review explores the impact of hyperglycemia on immune cells while providing greater insight into the molecular mechanisms of high glucose action and subsequent metabolic reprogramming of different immune cells. Furthermore, over-production of mitochondrial reactive oxygen species, formation of advanced glycation end products as a consequence of hyperglycemia and their downstream signalization in immune cells are also discussed. Since hyperglycemia in patients with type 1 diabetes mellitus might have an impact on immune-interventional treatment, the maintenance of a tight glucose control seems to be beneficial in patients considered for cell-based therapy.

## Introduction

Diabetes mellitus (DM) is a complex metabolic disorder resulting from immune-mediated destruction of insulin-producing cells in the islets of Langerhans (type 1 diabetes mellitus—T1D) or from the combination of insulin resistance and relative insulin deficiency (type 2 diabetes mellitus—T2D). Both types are associated with severe complications stemming from chronic hyperglycemia, the hallmark of diabetes mellitus (1). Hyperglycemia develops in T1D due to insufficient insulin production by pancreatic β cells. Transient episodes of hyperglycemia create an abnormal metabolic environment in various cell types leading to cell and tissue-specific metabolic reprogramming with subsequent macro- and microvascular complication in diabetic patients (2). Besides that, hyperglycemia also causes cumulative changes in long-lived macromolecules, which persist despite restoration of normoglycemia. This phenomenon was described as a metabolic memory, meaning that early high glucose environment is remembered by the cells (3).

Regarding the immune system, defects in the immune defense against miscellaneous pathogens were detected in patients with DM (4). Hyperglycemia is associated with decreased function of innate immunity such as lower complement levels, impaired chemotaxis, phagocytosis, and decrease in diapedesis of polymorphonuclear cells and monocytes/macrophages (5, 6). There is also considerable evidence that hyperglycemia contributes to the partial breakdown of peripheral tolerance (7), modulates patients' leukocyte profile (8), affects the function of antigen presenting cells (9), facilitates higher proinflammatory Th1/Th17 cells differentiation and suppresses regulatory T cells (Tregs) (10–12).

The adaptive immunity plays an essential role in the pathogenesis of T1D, particularly due to the imbalance between the autoaggressive effector T cells and Tregs (13, 14). In recent years, huge effort has been undertaken to suppress the inadvertent immune response in T1D. Several clinical trials and *in vitro* studies focused on cell-based therapy were launched with the goal to directly modulate the autoimmune destruction process of pancreatic β cells and to regenerate lost islets (15–18). Tolerogenic dendritic cells (tolDCs) and Tregs especially represent a new promising therapeutic strategy, either alone or in combinatorial therapies. Next, human stem cell (SCs) therapy represent another therapeutic approach for both inducing tolerance and islet cell regeneration (19). Current status of cell-based therapy is summarized in Table 1. However, little is known about the impact of the patient's glucose level on the potential cell-based vaccine's functional characteristics and efficacy. The initial immune cells isolated from hyperglycemic patient for the vaccine generation could exhibit different properties compared to those ones from euglycemic patients. Thus, the subsequent cell-based vaccine may exhibit different tolerogenic properties than in euglycemic subjects and the autoimmune destruction process in pancreas might be more difficult to suppress in patients with suboptimal glycemic control.

**Table 1:** Clinical studies (completed and with published results) for T1D treatment based on cells with regulatory properties including Tregs, tolerogenic DCs, and some examples of SCs.

	**Tregs**		**DCs**	**SCs**				
Trial ID	NCT01210664	ISRCTN06128462	NCT00445913	NCT01068951	NCT01374854	NCT00305344	NCT01350219
Cell definition	CD4^+^CD127^lo/−^CD25^+^ Polyclonal Tregs	CD4^+^CD25^high^CD127^−^ Tregs	Immunoregulatory DCs	Autologous MSCs	Allogeneic UC-MSCs plus autologous BM-MNC	Autologous Umbilical Cord Blood Transfusion	Cord blood-derived multipotent SCs	Adipose tissue-derived MSC-differentiated insulin-secreting cells plus BM-derived HSCs
Method of generation	Autologous Tregs isolated from the peripheral blood, expanded with anti-CD3/anti-CD28 beads in the presence of IL-2 and AB serum for 14 days	Autologous Tregs isolated from the peripheral blood, expanded with anti-CD3 and anti-CD28 antibodies, IL-2 and autologous serum for 7–14 days	Autologous DCs generated ex vivo from monocytes, modified using antisense oligonucleotides targeting primary transcripts of costimulatory molecules CD40, CD80 and CD86	MSCs aspirated from iliac crests and generated in growth media supplemented with lysed human platelets	Umbilical cord Wharton's jelly-derived MSCs generated in growth media supplemented with lysed human platelets; BM-MNCs aspirated from iliac crests	Umbilical cord blood as a source of immunomodulatory cells	In the Stem Cell Educator, lymphocytes separated from a patient's blood are briefly co-cultured with adherent CB-SCs and then returned to the patient	MSCs generated from adipose tissue, cultured for 10 days and further differentiate into insulin-secreting cells for 3 days; HSCs generated from BM
Application route	Intravenously	Intravenously	Intradermal (peri-umbilical region)	Intravenously	Infusion through pancreatic artery	Intravenously	Intravenously	Infused into portal circulation, thymus and into subcutaneous tissue
Cell number	0.05 × 10^8^, 0.4 × 10^8^, 3.2 × 10^8^, or 26 × 10^8^	10 or 20 × 10^6^/kg b.w., or 30 × 10^6^/kg b.w.	10 × 10^6^	2.1–3.6 × 10^6^ autologous cells/kg	1 × 10^6^/kg UC-MSCs plus 106.8 × 10^6^/kg MNCs	–	–	0.38–6.6 × 10^4^/kg b.w. insulin-secreting cells plus 17.4–149 × 10^6^/kg b.w. HSCs
Treatment application	1	1 (12 patients) or 2 (6/12 patients; 6–9 months apart)	4 (2 weeks apart)	1	1	1	1 or 2 (second after 3 months)	1
Results	No significant changes in C-peptide level (stable C-peptide level in 7/14 patients), HbA_1c_ level and insulin use after 2-year follow up; transiently ↑Tregs	↑C-peptide levels (8/12 and 4/6 patients after the first and the second dose, respectively), ↓insulin requirements (8/12, 2 patients insulin-independent) after 1-year follow up and ↓insulin requirements (4/12) after 2-year follow up; transiently ↑Tregs, ↓serum IL-1 and TNF-α	Partial ↑C-peptide (4/7); transiently ↑B220^+^CD11c^−^ regulatory B cells during 1 year follow up	Preserved or even increased C-peptide AUC (after meal tolerance test) during 1-year follow-up	↑C-peptide AUC (105.7%), ↑insulin AUC (49.3%) ↓ fasting glycemia (24.4%), ↓HbA_1c_ (12.6%), ↓insulin requirements (29.2%) at 1-year follow-up	No metabolic improvement (C-peptide level, HbA_1c_ level, insulin requirements); ↑Tregs during 2-year follow-up	↑C-peptide levels (fasting as well as after meal tolerance test), ↓HbA_1c_, ↓insulin requirements 25–38%; ↑Tregs, ↑serum TGF-β during 40-weeks follow-up after 1 application; residual β cell function preserved; ↑naïve CD4^+^ T cells and CD4^+^ T_CM_ cells, ↓CD4/8^+^ T_EM_ cells during 1-year follow-up after 2 applications (only patients with some residual β cell function)	↑ C-peptide levels, ↓Hb1_Ac_ levels and insulin requirements (all patients); ↓serum GAD antibody levels
References	(20)	(17, 21, 22)	(16)	(23)	(24)	(25, 26)	(27, 28)	(29)

*↑ Increase; ↓ Decrease; Treg, regulatory T cells; DCs, dendritic cells; IL, interleukin; BM, bone marrow; HbA_1c_, glycated hemoglobin A1c; TNF, tumor necrosis factor; SCs, stem cells; MSCs mesenchymal stem cells; UC-MSCs, umbilical cord mesenchymal stromal cells; BM-MNC, bone marrow mononuclear cell; AUC, area under curve; TGF, tumor growth factor; T_CM_, central memory T cells; T_EM_, effector memory T cells; HSCs, hematopoietic stem cells; GAD, glutamic acid decarboxylase*.

This review will focus on the regulating effect of hyperglycemia on immune cells, with a particular emphasis on tolerogenic DCs, T cells and Tregs. In addition, we will more specifically explore the subsequent changes in immune cell metabolism, initiation of alternative metabolic pathways such as the advanced glycation pathway, the process of advanced glycation end products (AGEs) formation and their molecular signaling in different immune cells.

## Effect of High Glucose Level on Different Signaling Pathways Via Reactive Oxygen Species and Advanced Glycation end Products

Hyperglycemia is associated with increased oxidative stress caused by over-production of nicotinamide adenindinucleotide (NADH) and mitochondrial reactive oxygen species (ROS), that inhibit glucose metabolism via glycolysis and tricarboxylic acid (TCA) cycle and consequently activate alternative glucose metabolic pathways including polyol pathway and hexosamine biosynthetic pathway (HBP). All these alternative pathways lead to increased ROS production, thus completing the vicious circle of cellular oxidative stress. Hyperglycemia-induced activation of protein kinase C (PKC) isoforms also strongly contributes to cellular and tissue damage by induction of proinflammatory gene expression and further ROS increase (30–32).

**Figure 1:**
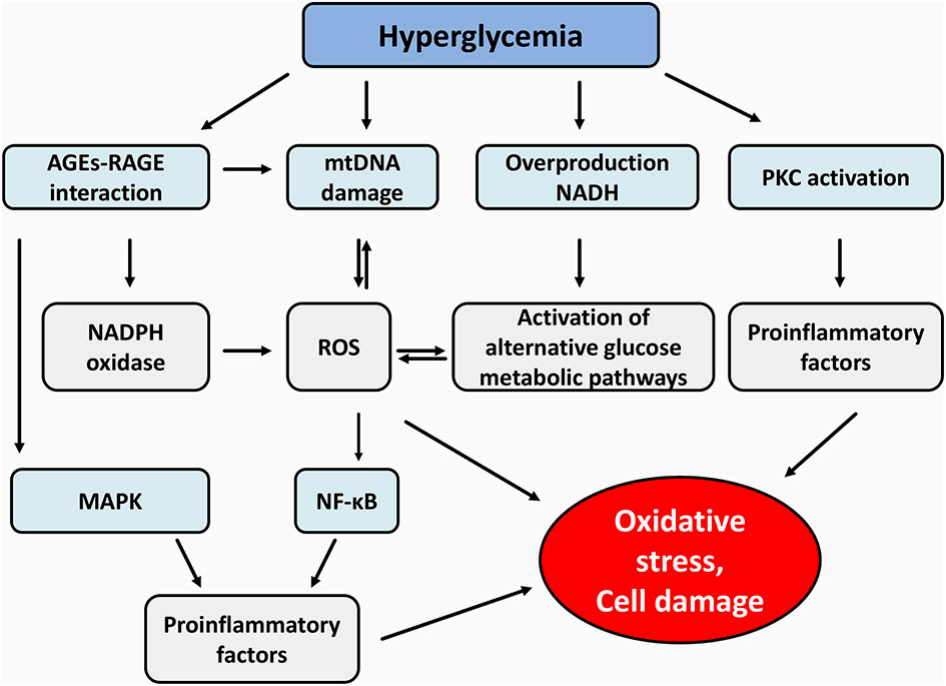
Effect of hyperglycemia and advanced glycation end products on different signaling pathways. AGEs, advanced glycation end products; RAGE, receptor for advanced glycation end product; mtDNA, mitochondrial DNA; NADH, nicotinamide adenine dinucleotide; PKC, protein kinase c; NADPH, nicotinamide adenine dinucleotide phosphate; ROS, reactive oxygen species; MAPK, mitogen-activated protein kinase; NF-κB, nuclear factor-κB.

Last but not least, the formation of advanced glycation products (AGEs) is an equally important mechanism in the processes of tissue-damaging effects of hyperglycemia. AGEs are heterogeneous compounds arising from irreversible non-enzymatic glycation of proteins, nucleic acids and lipids (33). One of the major products is glycated hemoglobin (HbA1c) that has been used as a biomarker for diabetes because it reflects long-term glycemia (34). Formation of AGEs occurs in several steps. First, the condensation of carbonyl group with a free amino group of proteins forms Schiff bases, which transform to more stable covalently bound Amadori products. The final irreversible products of glycation—AGEs arise from slow Amadori product rearrangements (35). In diabetic patients, chronic hyperglycemia-driven accumulation of AGEs and its cognate receptor for advanced glycation end-product (RAGE) are involved in the pathogenesis of both micro and macrovascular complications (36). Indeed, the formation of AGEs interferes with cell integrity by irreversible glycation of various proteins. Furthermore, signaling via RAGE-mediated pathways increases ROS production via the activation of an NADPH oxidase system which contributes to further mitochondrial protein damage and DNA damage (37). RAGE consists of 5 domains (three extracellular, one transmembrane and one intracellular domain) (38). The intracellular domain is important for the activation of the transcriptional factor nuclear factor-κB (NF-κB), which leads to expression of growth factors, cytokines and RAGE itself in miscellaneous cells (39). In endothelial cells, downstream signaling via AGEs-RAGE receptor complex activates the signaling pathways of glycogen synthase kinase 3β (GSK3β), p38 mitogen-activated protein kinase (MAPK), extracellular signal-regulated kinase 1 and 2 (ERK1/2), c-Jun amino-terminal kinase (JNK) and NF-κB, all of which lead to endothelial cell dysfunction and diabetic vascular disease. Moreover, AGEs play an important role in β-cell failure by activating NADPH oxidase with a consequence of increased ROS generation and induction of β-cell apoptosis through the PKCβ2 pathway (40, 41) (Figure 1).

RAGE receptors are expressed on the surface membrane of various immune cells and can be found also in the cytosol. Several findings have been recently reported that AGEs-RAGE mediated signaling causes the induction of dendritic cell (DCs) maturation, Th1/Th17 polarization from naïve CD4^+^ T cells, activation and maturation of B cells and the reduction of the suppressor function of Tregs (9, 42–44).

## Effect of High Glucose Level on Tolerogenic Dendritic Cells

Dendritic cells, the most potent antigen-presenting cells (APC), are crucial for the induction of the pathological process in autoimmune DM. TolDCs represent immature or semi-mature DCs capable to promote immune tolerance by various mechanisms such as induction of autoreactive T cell hyporesponsiveness, anergy, or apoptosis and by induction of various types of T and B regulatory cells. These essential properties determine tolDCs as a new promising strategy for prevention of autoimmune DM in the subjects who are at risk or even for treatment of T1D. A human phase I trial of autologous monocyte-derived tolDCs in T1D has been completed (16, 45) and another trial based on proinsulin-loaded tolDCs has been recently opened (46).

Dendritic cell immune-metabolic state dictates the balance between immunity and tolerance. Immature DCs utilize oxidative phosphorylation (OXPHOS) and fatty acid oxidation (FAO) as their main energy sources, while DC maturation is followed by a pronounced switch to aerobic glycolysis (47, 48). TolDCs show a distinct metabolic profile. Generally, they exhibit catabolic and highly energetic profile favoring OXPHOS and FAO; however, metabolic plasticity can be observed regarding the protocol used for tolDCs generation (49, 50). Vitamin D3 (VitD3) and its derivatives, immunosuppressive drug dexamethasone (DEX) or Vit/DEX combination are widely used for tolDC generation (51, 52). Malinarich et al. reported high mitochondrial activity, increased OXPHOS, a shift in redox state and high glycolytic capacity as the metabolic signatures of VitD3/DEX generated tolDCs. In this study, inhibition of FAO caused increased expression of maturation markers on tolDCs and partially restored their T cell stimulatory capacity, suggesting their dependence on FAO (53). Ferreira et al. showed that VitD3–induced tolDCs exhibited increased glucose metabolism, high mitochondrial activity and elevated OXPHOS profile with glucose as the main oxidative fuel compared to mature DCs. Interestingly, PI3K/Akt/mTOR driven glycolysis, but not glucose oxidation, was crucial for the maintenance of tolerogenic phenotype of VitD3-modulated tolDCs (54). Next, we documented in our study that increased glycolysis contributes to the stability of VitD2/DEX generated tolDCs in the inflammatory environment (55). Another study pointed out the profound metabolic changes toward increased OXPHOS and lipid metabolism while reducing amino acids and fatty acid synthesis in VitD3/DEX generated tolDCs compared to mature DCs (50). Such catabolic metabolic reprogramming with nutrient deprivation supports regulatory T cell induction (56).

The studies assessing the potential effect of hyperglycemia, oxidative stress and AGEs in diabetic patients on tolDCs-based vaccine generation and its functional properties are limited. In our study we analyzed the impact of the metabolic state on functional properties of tolDCs generated from patients with T1DM, aiming to investigate the potential group of patients targeted for tolDCs immunotherapy. We showed that monocyte-derived tolDCs generated by DEX and VitD2 displayed better regulatory properties when prepared from the group of T1D patients with tight glucose control. TolDCs generated from T1D patients with a suboptimal level of glycated hemoglobin (HbA1c) exhibited down-regulation of immunoregulatory molecules PD-L1 and IL-T3 in comparison with tolDCs prepared from patients with optimal HbA1c level. Tolerogenic effect of tolDCs was better in normoglycemic patients in terms of more efficient suppression of Th1 and Th17 related cytokines production. Indeed, autologous primary culture of T cells and tolDCs from patients with optimal glucose control showed lower supernatant concentration of Th1-related cytokines IFN-γ and TNF-α and Th-17 related cytokine IL-17A, IL-23 and IL-9 and upregulated levels of IL-10 compared to cultures with control DCs. However, in patients with high level of HbA1c, we detected significantly lower levels of IFN-γ, TNF-α, and IL-9, but not IL-17A and IL-23 in comparison to control DCs. Overall, the ability of tolDCs to induce hyporesponsiveness of autologous T cells accompanied by a reduction of Th1 and Th17 cytokines was better in patients with tight glucose control. Importantly, improvement of glycemic control in T1D patients restores the ability of tolDCs to tolerize their autoreactive T cells (12).

One of the potential explanations for better effectiveness of DEX/VitD2-generated tolDCs from patients with tight glucose control is that hyperglycemia might attenuate the expression or function of vitamin D receptor (VDR) on monocytes used for generation of tolDCs. Indeed, hyperglycemia inhibited VDR expression in human vascular smooth cells, induced glycosylation of VDR in human monocytes and macrophages due to hexosamine pathway activation and interacted with VDR to impair its DNA binding and function. Thus, the high glucose level might exacerbate the function of VitD as a crucial tolerogenic agent indispensable for IL-T3 and PD-L1 expression on tolDCs (57–61). Next, high glucose, ROS, and AGEs have been previously shown to have an effect on *in vitro* DC generation from blood monocytes. Indeed, high glucose impaired *in vitro* differentiation of monocytes from healthy donors into DCs by inducing ROS, activating Wnt/β-catenin pathway and p38MAPK (62). Moreover, AGEs treatment led to persistent NF-κB activation and abnormal NF-κB function observed in T1D monocytes (63, 64). As Dex or Vitamin D receptor agonists have been described to generate tolDCs through NF-κB down-regulation, it is possible that well-controlled patients have a better capacity to overcome sustained hyperglycemia driven NF-κB activation in the process of tolDCs generation.

Once the immature or semimature tolDCs are applied to the patients' body, they will experience proinflammatory environment and high glucose milieu. Although the stability of various tolDCs in the proinflammatory environment is well documented, the data assessing the effect of high glucose are scarce (55, 65, 66). Regarding the effect of high glucose on immature DCs, short-term (24–48 h) high glucose treatment of monocyte-derived immature DCs generated from healthy donors accelerated the expression of co-stimulatory molecules, such as CD83 and CD86, and induced proinflammatory cytokine profile with up-regulation of IL-6 and IL-12 while the level of IL-10 was diminished (9, 67). Additionally, high glucose enhanced up-regulation of several DCs scavenger receptors, probably via increased production of intracellular ROS, and the activation of p38 MAPK pathway (67). Other studies demonstrated that AGE-modified serum molecules augmented the capacity of DCs to stimulate T cell proliferation and T cell cytokine secretion possibly through the up-regulation of RAGE on DCs. The subsequent activation of MAPK pathways and NF-κB was crucial for this phenomenon (68, 69). Buttari et al. documented that polyphenolic antioxidant resveratrol prevented the immature DC maturation, IL-12, IL-1β, TNF-α production and diminished the allostimulatory capacity of AGEs-treated DCs via abrogation of MAPK and NF-κB activation (70). Overall, these findings highlight the role of ROS, MAPK, and NF-κB as signaling molecules mediating the activating effect of high glucose in monocyte-derived DCs. Thus, the possibility exists, that tolDCs activated by high glucose conditions or AGEs might modify their tolerogenic profile into more matured and less potent phenotype due to the augmented DCs activation, presence of maturation markers and favorable cytokine profile. However, further studies are needed to fully elucidate the effect of high glucose levels, oxidative stress, and ROS on the stability of tolDCs.

**Figure 2:**
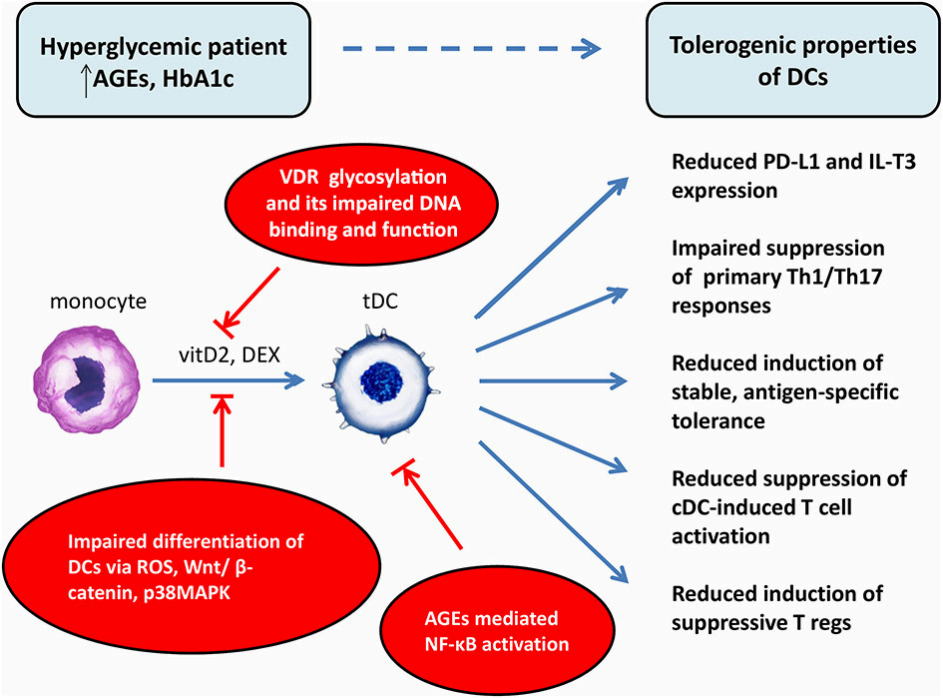
Potential explanations of impaired effect of tolerogenic DCs in vaccine generated from patients with chronic hyperglycemia. DCs, dendritic cells; cDC, control dendritic cells; tDC, tolerogenic dendritic cells; T regs, T regulatory cells; AGEs, advanced glycation end products; HbA1c, glycated hemoglobin; VDR, vitamin D receptor; vitD2, vitamin D2; DEX, dexamethasone; ROS, reactive oxygen species; NF-κB, nuclear factor-κB; Wnt, wingless/integrated signaling pathway; p38MAPK, p38 mitogen-activated protein kinases; PD-L1, programmed death-ligand 1; IL-T3, immunoglobulin-like transcript 3.

So far, we can just speculate whether and how hyperglycemia can modulate bioenergetics and metabolism of tolDCs once they experience hyperglycemic conditions in T1D patients. As discussed above, hyperglycemia drives dysregulation of glycolysis as well as mitochondrial TCA cycle leading to mitochondrial respiratory chain complex dysfunction and the production of increased levels of ROS. Moreover, hyperglycemia driven AGEs formation and hexosamine biosynthetic pathway activation participate on the post-translational modification of protein subunits of mitochondrial electron transport chain (ETC) complexes leading to impaired mitochondrial function (31). Given the critical role of metabolic pathways in sustaining tolDCs function, exposure to hyperglycemia might affect the behavior of tolDCs and the outcome of the tolDCs therapy. On the other hand, some data suggest that once the metabolic reprogramming takes place in particular tolDCs, they sustain their tolerogenic phenotype independently of the actual glycemia level (Figure 2).

## Effect of High Glucose Level on T-cells

Metabolically aberrant microenvironment caused by hyperglycemia in cooperation with chronic low-grade inflammation might affect T cell metabolism and immunological phenotype. Naïve T cells are in metabolically dormant state and primarily rely on OXPHOS and FAO to meet their bioenergetics and biosynthetic demands (71, 72). However, upon activation naïve T cells rapidly switch to high rates of glycolysis, glutaminolysis, and OXPHOS for cell growth and proliferation (73). Hyperglycemia drives increase in glycolysis in activated T cells thus leading to robust IFN-γ production by preventing glycolytic enzyme glyceradehydphosphate dehydrogenase (GAPDH) from binding to and inhibiting the translation of IFN-γ mRNA (2, 74) and thus driving inflammation in a non-antigen specific manner. Moreover, hyperglycemia-driven oxidative stress exerts a global effect on T cells as shown by T cell hyperresponsiveness (75), induction of proinflammatory Th17 cells (10, 11) and diminished regulation of IL-7-mediated T cell survival and homeostatic expansion (76). Thus, it is plausible that “pre-activated” phenotype of T-cells detected in diabetic subjects (75) may affect the ability of immunoregulatory cell vaccine to induce the generation of antigen-specific tolerance in T cells.

In our study we reported that DEX/VitD2-generated tolDCs from patients with euglycemia, but not hyperglycemia, markedly reduced not only primary Th1/Th17 responses, but also mature DC-induced proinflammatory IFN- γ, IL-17, TNF-α, and IL-9 production. Suppressive effect of tolDCs on mature DC-induced cytokine production correlated negatively with HbA1c level. Interestingly, T cells form hyperglycemic patients exhibited higher basal Th1/Th17 cytokine production and higher basal proliferation (antigen- independent) alongside with lower responses to antigen stimulation in primary cultures (12). The similar observation was reported on IL-10/TGF-β-induced tolDCs from T1DM patients (15, 77), where tolerance induction was dependent on the current activation state of T cells in each patient. In Segovia-Gamboa's study, antigen-specific T cell tolerance was lost in patients with high homeostatic T cell proliferation and low autoantigen-specific T cell response and the magnitude of T cell suppression inversely correlated with the level of HbA_1c_ (15). These data suggest that hyperglycemia reduces the ability of tolDCs to induce stable antigen-specific T cell tolerance by making autoreactive T cells more activated and thus less prone to be tolerized.

Nevertheless, the mechanism of increased T cell activation/proliferation in hyperglycemic diabetic patients is not fully understood. It has been assumed that hyperglycemia driven oxidative stress, AGEs formation, and AGEs-RAGE interaction is one of the possible cause as RAGE ligation affects T cell activation and controls T cell differentiation (44, 78). Mechanistically, Kumar et al. showed that hyperglycemia drives the expression of proinflammatory cytokines and chemokines, especially IL-6 and IL-17 family members transcriptionally through oxidative stress and NF-κB activation via PKC and p38MAPK pathway in blood lymphocytes from diabetic subjects (11). Another study reported that AGEs dose-dependently promoted RAGE expression and induced differentiation of naïve CD4+ T cells into Th1/Th17 phenotype via down-regulation of transcription factor peroxisome proliferator-activated receptor gamma (PPAR-γ). RAGE knock-down abolished the AGEs-induced Th1/Th17 differentiation (10). Interestingly, RAGE is constitutively present intracellularly in both CD4+ and CD8+ T cells from diabetic subjects compared to healthy donors where RAGE was found to be expressed only following TCR activation. Moreover, RAGE+ T cells in diabetic patients display proinflammatory gene expression profile and express high levels of IL-17A. Elevated RAGE expression in diabetes is most likely due to the abundant overproduction of RAGE ligands including AGEs in diabetic patients (79) since it was detected on T cells in diabetic patients but not in T cells from patients with other autoimmune diseases such as Sjogren's syndrome and rheumatoid arthritis (80). Despite no correlation of RAGE expression with the level of glycemia in this study, the abundant overproduction of RAGE ligands in diabetic patients supports the hypothesis that glycated molecules forming AGEs could be contributing factor for exaggerated activation of T cells. On the other hand, higher expression of intracellular RAGE was shown also in high-risk euglycemic relatives (81). Thus, the direct impact of primary impaired T cell activation pathway in autoimmune diabetes cannot be ruled out.

Chronic hyperglycemia-induced “pre-activated” T cells phenotype was presented also in the mouse with streptozotocin-induced diabetes. Naïve T cells from hyperglycemic mice showed functional marks of antigen-experienced T cells with increased Th1, Th2, and Th17 cytokine production and higher proliferation upon TCR stimulation compared to euglycemic control, despite having the low CD44 expression. Simultaneously, they exhibited considerable chromatin decondensation which resulted in the facilitation of the transport of transcriptional factors to the DNA. These findings could be a consequence of inner modulation of TCR downstream regulators in high glucose milieu and are likely to be RAGE-dependent as RAGE deficiency reversed the hyperactivated phenotype of T cells in hyperglycemic mice. Of particular interest, all hyperglycemia-induced changes persisted after adoptive transfer into euglycemic hosts (75). This points to the phenomenon of metabolic memory and partly explains why the activating phenotype of T cells may persist even after glucose normalization.

## Effect of High Glucose Level on T Regulatory Cells

Both naturally occurring Tregs arising from thymus (nTregs) as well as induced T regs (iTregs) developing in the periphery are the key negative regulators of the immune response and play a crucial role in the maintenance of the peripheral tolerance. Administration of *ex vivo* expanded Tregs has been considered as another interesting strategy for the treatment of T1D. Early clinical studies utilizing polyclonally expanded autologous CD4+CD25+FoxP3+ Tregs demonstrated the safety and feasibility of this approach and supported their therapeutic potential for inhibition or delay of the destruction of pancreatic β cells in T1D patients (17, 20).

Recent studies utilizing more precise Tregs markers such as low CD127 expression or the selective demethylation of certain regions of the Foxp3 locus (82) show that T1D patients appear to have normal T regs frequencies compared to healthy donors, but they are often poorly functioning (83–85). The lower suppressive capacity of Tregs from T1D patients, their phenotypic shift accompanied by decreased production of anti-inflammatory cytokines IL-10, IL-35 and TGF-β and increased production of inflammatory cytokines IFN-γ and IL-17 (84, 86–89), unstable FoxP3 expression (90), and higher susceptibility to apoptosis were documented (91–95).

Whether the functional defect stems from the inner modulation of Tregs population in a subject with an autoimmune disease or whether it is partly caused or deepened by hyperglycemia remains to be clarified (96). Glycolysis was shown to orchestrate the generation and suppressive function of human iTregs by controlling of FoxP3 splicing variants through the glycolytic enzyme enolase-1 (97) that can bind DNA and regulate gene expression. Glycolysis was also shown to affect expression of Tregs' cell inhibitory and regulatory molecules CTLA-4, PD-1, GITR, CD37, and CD71 (97). Thus, impaired glycolysis observed in T1D might participate on defective induction/function of Tregs (98). Next, hyperglycemia contributes to increased expression of IL-6 and TGF-β (11, 99) that synergistically down regulate FoxP3 at the post-translational level by promoting FoxP3 protein degradation in Tregs (100).

The negative effect of hyperglycemia on Tregs phenotype and function was observed also in streptozotocin (STZ) induced hyperglycemic mice, where long-term hyperglycemia changed the phenotype of CD4+CD25+FoxP3+ Tregs into activated/memory phenotype with lower CD62L, CD45RB, GITR, and higher CTLA-4 expression and reduced their suppressive effect on effector T cells proliferation. These properties were unambiguously reversed by insulin administration (101). In human studies from healthy donors, glucose-induced AGEs also reduced the suppressive function of Tregs and decreased the ratio of Tregs/Th17 cells (10). In T1D patients, the frequency of Tregs correlated negatively with the level of HbA_1c_ (102–104).

Next, Tregs defective function and number might result from defective signaling from dendritic cells which under hyperglycemia might exhibit weaker tolerogenic potential. Indeed, in our study, co-cultures of naïve T cells and antigen-loaded tolDCs from patients with high HbA_1c_ levels led to the induction of lower levels of proliferating CD4+CD25^high^CD127^low^FoxP3+ Tregs producing low IL-10 levels compared to euglycemic patients. Of a great interest, Tregs expanded by tolDCs from patients with high HbA_1c_ levels exhibited weaker suppressive abilities (12). In this case, it seems that reduced surface expression of ILT-3 and PD-L1 on dexamethasone/VitD2-generated tolDCs from poorly compensated T1D patients might play a role in defective Tregs induction and function (12).

From the present clinical studies is not clear whether the glycemia level on diabetic subjects influents the quality of resulting vaccine or not. Okubo et al. demonstrated in their study that T1D patients with tight glucose control (based on the HbA1c level) showed higher percentage of activated Tregs (CD4+CD25+FoxP3+CD45RA+) in peripheral blood and lower percentage of resting Tregs (CD4+CD25+FoxP3+CD45RO+). As activated Tregs are the most suppressive Tregs, their higher percentage in primary culture could increase suppressive effect of the resulting vaccine in patient with optimal glucose control (105).

## Effect of High Glucose Level on Stem Cell-Based Therapy

Stem cells (SCs) are multipotent, self-renewing cells, with anti-inflammatory and immunomodulatory properties. Implantation of SCs to T1D patients might regulate ongoing autoimmune process by inhibition of T and B cell activation, DCs differentiation and NK cell activity, while induction of T cell anergy and T regs generation (106, 107). Moreover, SCs can differentiate into insulin-producing cells and revitalize the damaged pancreatic β-cells. Sources for SCs therapies in diabetes mellitus can be multiple, including embryonic stem cells (ESCs), cord blood stem cells, induced pluripotent stem cells (iPSCs), and adult stem cells derived from adult tissues. Among all kinds of SCs, mesenchymal stem cells (MSCs) derived from the bone marrow or other sources, have been shown as an interesting viable approach for treatment of T1D and tested in human clinical studies aiming to prevent or arrest the onset and progression of T1D, inhibit β-cell destruction, and restore glycometabolic and immune homeostasis. Despite the favorable results indicating potential efficacy of MSCs to preserve β-cell function in some T1D patients demonstrated by the higher C-peptide level, decreased insulin doses and improved HbA_1c_ levels, there are still doubts on the long-term effectiveness of MSCs for the management of T1D (108, 109).

Regarding the hampering effect of hyperglycemia on MSCs therapies, several studies reported that hyperglycemic state and underlying defective microenvironment in diabetic patients impair SCs function. Indeed, high glucose can induce senescence of MSCs via Akt/mTOR signaling (110). Next, hyperglycemia and subsequent oxidative stress triggered by high glucose or chronic RAGE signaling might have a large negative effect on the differentiation, proliferation and regeneration capability of MSCs by affecting Wnt/β-catenin pathway, PI3K/Akt pathway, MAPK signaling, PKC pathway or micro RNA expression (111–116). Study by Kornicka et al. documented that MSCs isolated from patients with T2D exhibited increased apoptosis, autophagy, ROS accumulation and mitochondria deterioration (117). In another study, hyperglycemic conditions in embryos hindered differentiation of human embryonic stem cells by changing their histone methylation pattern leading to pancreatic malfunction (118). Thus, it seems that hyperglycemic conditions in T1D patients might limit therapeutic potential of MSCs-based therapies.

## Conclusions

In summary, new emerging evidence suggests that chronic hyperglycemia in patients with diabetes markedly influences their immune system. The rising incidence of T1D calls for new therapeutic options and the immune suppression strategy based on the tolerogenic DCs or T regs vaccines seems to be very promising. The purpose of our review was to summarize the effect of hyperglycemia on various cells of adaptive immunity aiming to clarify the link between the patient's metabolic status and different efficacy of immune interventional treatment. One of the important mechanisms mediating the effect of chronic high glucose level is AGEs formation and their signaling through a specific receptor—RAGE and other groups of scavenger receptors. In essence, AGEs-RAGE interaction leads to activation of transcription factor NF-κB and ROS generation via diverse downstream signaling pathways including MAPK kinase signaling. Therefore, it is believed that this mechanism could contribute to hyperglycemia-induced proinflammatory environment in the body.

For the purpose of cell-based vaccine, tolDCs are generated in autologous system from monocytes of the peripheral blood of patients. Thus, the different status of patient's metabolic control may provide a different input for the cell-based vaccine resulting in a different final quality. Taking into account the metabolic memory phenomenon together with the fact, that even transient episodes of hyperglycemia were associated with epigenetic changes in several cells including progenitor cells (119), the very early tight glucose control seems to be essential for adequate effect of resulting tolerogenic vaccination therapy. In fact, resistance to a further maturation stimulus such as proinflammatory environment and high glucose milieu is a prerequisite for tolerogenic DC clinical application, since a potential transformation of immature or semi-mature DCs into fully mature DCs would lead to the acquisition of the capacity to promote immunogenic instead of protective immune responses and thereby to exacerbation of patient's autoimmune condition. Similarly, stem-cell based vaccines might lose their immunomodulatory potential by facing hyperglycemia conditions at the time of cell administration. Controversially, our previous study showed that alteration of some immune cells (specifically tolDCs) caused by hyperglycemia may be reversed after improvement of glycemic control (12). Thus, the equal importance should be considered for optimal vaccine timing, preferably in the period of ideal metabolic control.

Not only patient's metabolic status, but also appropriate manufacturing protocols for generation of cell-based vaccines, especially with regard to glucose concentration, has to be managed as the applied culture media components may influence the tolerogenic properties of generated immune cells. Indeed, concentration of glucose in culture media above 10 mM are analogous to hyperglycemic conditions in diabetic patients and the cells growing under high-glucose conditions are modified by processes of glycation, glyoxidation, and subsequent oxidative stress. Several important media in bio-manufacturing contain diabetic levels of glucose supplementation (for example DMEM (Hi), GMEM, and IMDM media all contain 25 mM levels of D-glucose). For tolDCs manufacturing and/or T regs expansion Cell Gro GMP medium or X-VIVO 15 medium are widely used, however the precise glucose concentration in those media is unknown and should have been tested in house to avoid hyperglycemic culture conditions (21, 120, 121).

The final question remains, how to circumvent the hyperglycemia issues in cell-based therapy for T1D. Based on the data mentioned above, special attention should be paid on managing patient's metabolic status. Precise adjustment of insulin regimen or conversion to insulin pump for tight glucose controlling seems to be the first step in consideration of cell-based therapy. Managing manufacturing process for generation or expansion of immune cells (appropriate culture conditions) and adjusting precise timing of cell-based vaccine application (euglycemia in patients) are equally important. Furthermore, it remains to be tested whether application of oxidative stress-reducing compounds such as resveratrol during *in vitro* immune cells generation /or expansion might in consequence improve the possible dysfunctionality of patient's immune cells and the efficacy of cell-based therapies (122). Regarding MSCs, considering allogeneic healthy donors as a source of MSCs for cell based therapies in diabetic patients might be another option.

To sum up, the precise effect of hyperglycemia on immune cells is not fully clear. More detailed studies should be performed to distinguish the genuine effect of high glucose from the changes in immune cells caused by autoimmune disease itself in T1D patients. Nevertheless, based on the recent knowledge, hyperglycemia in diabetic patients may have an impact on immune-interventional treatment and maintaining of a tight glucose control seems to be beneficial in patients considered for cell-based therapy.

## Author Contributions

AG and LP-J wrote the manuscript and KD and RŠ edited while adding additional insights. The final version was proofread and edited by RS.

### Conflict of Interest Statement

LP-J, KD, and RS are named inventor in a related patent, Tolerogenic Dendritic cells, Methods of Producing the Same, and Uses Thereof PCT/EP2015/074536 which describes methods for the preparation of stable semi-mature tolDCs. The remaining author declares that the research was conducted in the absence of any commercial or financial relationships that could be construed as a potential conflict of interest.
